# Regional Brain Volumes Associated With Depression in the National Alzheimer’s Coordinating Center Uniform Data Set

**DOI:** 10.1093/geroni/igaa057.1196

**Published:** 2020-12-16

**Authors:** Shanna Burke, Tan Li, Adrienne Grudzien, Christopher Barnes, Kevin Hanson, Steven DeKosky

**Affiliations:** 1 Florida International University, Miami, Florida, United States; 2 University of Florida, Gainesville, Florida, United States; 3 University of Florida College of Medicine, Gainesville, Florida, United States

## Abstract

Depression has been associated with greater risk of Alzheimer’s disease (AD), and existing research has identified structural differences in brain regions in depressed subjects compared to healthy samples, but results have been heterogeneous. We sought to determine the effect of depression on regional brain volumes by cognitive and APOE e4 status. Secondary analysis of the National Alzheimer’s Coordinating Center (NACC) Uniform Data Set was conducted using complete MRI data from 1,371 participants (mean age: 70.5; SD: 11.7). Multiple linear regression was used to estimate the adjusted effect of depression (via the Neuropsychiatric Inventory Questionnaire) on regional brain volumes through measurement of 30 structural MRIs. Depression in the prior two years was associated with lower total brain, cerebrum,, and gray matter volumes and greater total brain white matter hyperintensities (p<.05). Greater volumes were also observed in all ventricular volume measures. Lower mean volumes were observed in six additional frontal lobe and parietal lobe cortical regions. Alternately, depression antecedent to the past 2 years correlated only with occipital lobe gray matter volumes (right, left, total). Our findings suggest that depression in the prior two years is associated with atrophy across multiple brain regions and related ventricular enlargement, even after controlling for intracranial volume and demographic covariates. The duration of depression influences results, however, as depression prior to 2 years before assessment was correlated with significantly fewer and different regional brain volume changes.

